# Spatial and temporal characteristics of drought and its correlation with climate indices in Northeast China

**DOI:** 10.1371/journal.pone.0259774

**Published:** 2021-11-18

**Authors:** Yuan Yue, HaiFeng Liu, XiuXiang Mu, MengSheng Qin, TingTing Wang, Qi Wang, YaQiong Yan

**Affiliations:** 1 Meteorological Observatory of Jilin Province, Changchun, China; 2 Yangzhou Meteorological Bureau, Yangzhou, China; 3 Institute of Meteorology Science of Jilin Province, Changchun, China; 4 Suqian Meteorological Bureau, Suqian, China; Universiti Teknologi Malaysia, MALAYSIA

## Abstract

The spatial and temporal characteristics of drought in Northeast China are investigated, using monthly meteorological data from 140 stations over the period 1970–2014. The study area was divided into three regions using hierarchical cluster analysis based on the precipitation and potential evapotranspiration data. The standardized precipitation evapotranspiration index (SPEI) was calculated for each station on 3-month and 12-month time scales. The Mann-Kendall (MK) trend test and Sen’s slope method were applied to determine the trends for annual and seasonal SPEI time series. Periodic features of drought conditions in each sub-region and possible relationship with large-scale climate patterns were respectively identified using the continuous wavelet transform (CWT) and cross wavelet transform. The results show mitigations in spring and winter droughts and a significant increasing trend in autumn drought. On the annual scale, droughts became more severe and more intense in the western regions but were mitigated in the eastern region. CWT analysis showed that droughts in Northeast China occur predominantly in 14- to 42-month or 15- to 60-month cycles. Annual and seasonal droughts have 2- to 6-year cycles over the three defined regions. Cross wavelet analysis also shows that the statistically significant influence of large-scale climate patterns (the Southern Oscillation, the Atlantic Multidecadal Oscillation, the Arctic Oscillation, and the Polar–Eurasian Pattern) on drought in Northeast China is concentrated in a 16- to 50-month period, possibly causing drought variability in the different regions. The Southern Oscillation, Polar–Eurasia pattern, and Arctic Oscillation are significantly correlated with drought on decadal scales (around 120-month period). The findings of this study will provide valuable reference for regional drought mitigation and drought prediction.

## Introduction

Drought is a natural hazard caused by a period of below-average precipitation in a given area. A drought can last for weeks, months, or years. The development of a drought is generally influenced by atmospheric circulation and the hydrological cycle [1, 2]. It is commonly thought that droughts are irregular in both spatial distribution and development. Damage caused by drought is cumulative and increases as the drought develops and it may persist over a considerable period of time, even after the drought ends. This feature of droughts led to them being described as *creeping* features [3]. Due to their creeping nature and the irregularity of their occurrence and duration, droughts are more difficult to identify and assess than other natural hazards, such as typhoons, severe rainstorms, or floods.

The importance of the correlation between local droughts and large-scale atmospheric circulation patterns has recently become better understood. The development of drought is closely related to the anomalies of atmospheric circulation patterns (which are represented by various circulation indices) such as the El Niño–Southern Oscillation (ENSO), the North Atlantic Oscillation (NAO), the Atlantic Multidecadal Oscillation (AMO), the Pacific Decadal Oscillation (PDO), the Polar–Eurasian teleconnection pattern (POL), the East Asian Trough Position Index (IEAT), and the Asian Zonal Circulation Index [4–7]. In Northern China, the warm phases of PDO correlate with drought and the cold phases with wet conditions [8]. Wang et al. [9] found possible correlations between large-scale climate patterns (ENSO and AMO) and drought in the Luanhe River basin over different time scales. Droughts in East Asia are often associated with large-scale atmospheric circulation rather than local climate systems [10, 11].

Global air temperature is increasing annually, relative to the 1880–2012 average temperature, in the range 0.65°C to 1.06°C; there has been an overall increase of 0.85°C in average annual temperature over the period 1880–2012 [12]. Global warming has resulted in increased occurrence of extreme climate events in recent years over different parts of the world [13, 14]. Thus it is to be expected that, worldwide, droughts occur more frequently and have become more severe in recent years [15, 16]. Droughts in China have increased significantly in number since the middle of the twentieth century [17–20]. Yu et al. [21] analyzed data for severe and extreme droughts across mainland China from 1951 to 2010. Their results show an increasing extreme drought trend since the late 1990s over most of China and an average increase in the total area affected by droughts of 3.72% per decade. Chen and Sun [22], also using SPEI, found that droughts had become more frequent and more severe across China since the late 1990s, mainly in Northern China.

Northeast China is a major grain producing area. It lies between 120° and 135° N and between 38° and 53° E and covers 78 730 000 km^2^. It is strongly affected by the East Asia monsoon (EAM) system. Droughts have become longer and more severe in the area due to global warming [23, 24]. Droughts affect grain production, so increasing drought poses a greater threat to agricultural development and puts future agricultural production at risk [25]. There has been little assessment of the effects of drought and the possible correlation between large-scale climate patterns and droughts in Northeast China. An analysis of drought in relation to atmospheric circulation in this area, using SPEI, will benefit agricultural development and improve the management of future drought risk.

Drought indices have been developed and used to identify and characterize droughts; they include the Palmer drought severity index (PDSI) [26]; the standardized precipitation index (SPI) [27];, the reconnaissance drought index (RDI) [28];, and the standardized precipitation evapotranspiration index (SPEI) [29]. SPEI has been widely used to characterize droughts in different geographical areas because it incorporates the two most important factors (precipitation and evapotranspiration) that simultaneously influence drought and because it effectively characterizes the effect of increased temperature on drought. The index is obtained by normalizing the difference between calculated precipitation and calculated potential evapotranspiration over some particular time scale. Flexibility in choice of time scale means that SPEI can be used to compare drought severity over different time scales and areas [7, 21, 30–34].

In this study, we used the Penman–Monteith (PM) equation in the calculation of SPEI. The Penman–Monteith equation incorporates humidity, air pressure, wind speed, and radiation which makes it more representative of physical conditions than the Thornthwaite equation [35] that was used in the original development of SPEI by Vicente-Serrano et al. [29]. The Penman–Monteith method has been applied to the study of drought in different regions [36, 37]. On the SPEI website, the PM method has become the default method for estimating PET in the calculation of SPEIbase V2.0. However, the calculator they made available still uses the Thornthwaite method so we used the FAO-56 Penman–Monteith method to calculate SPEI.

Cross wavelet analysis is widely and effectively used to study and test correlations between time series. It is a powerful tool and is used in climatology in order to identify forcing mechanisms between time series [38–40]. For example, Su and Li [41] analyzed possible connections between drought conditions and large-scale climate indices in Beijing and found significant correlations between drought and four circulation patterns (AO, ENSO, NAO, PDO). Such work suggests that cross wavelet analysis can be used to understand connections between large-scale climate patterns and drought conditions in this study.

The objective of this study is to systematically analyze the spatial and temporal characteristics of drought conditions over Northeast China by using SPEI at 3-month (SPEI-3) and 12-month (SPEI-12) time scales and the relationship between drought condition and different climate patterns. The results of this study can be used as reference for regional drought mitigation and prediction. The study area is first divided into smaller homogeneous regions using cluster analysis. Then temporal trends and periodic features of droughts in each region are identified using the Mann–Kendall (MK) trend test, followed by trend analysis using Sen’s slope estimator and wavelet analysis. Finally, cross wavelet analysis is used to investigate the possible relationship between individual drought variability and large-scale climate patterns.

## Material and methods

### Data

**Fig 1:**
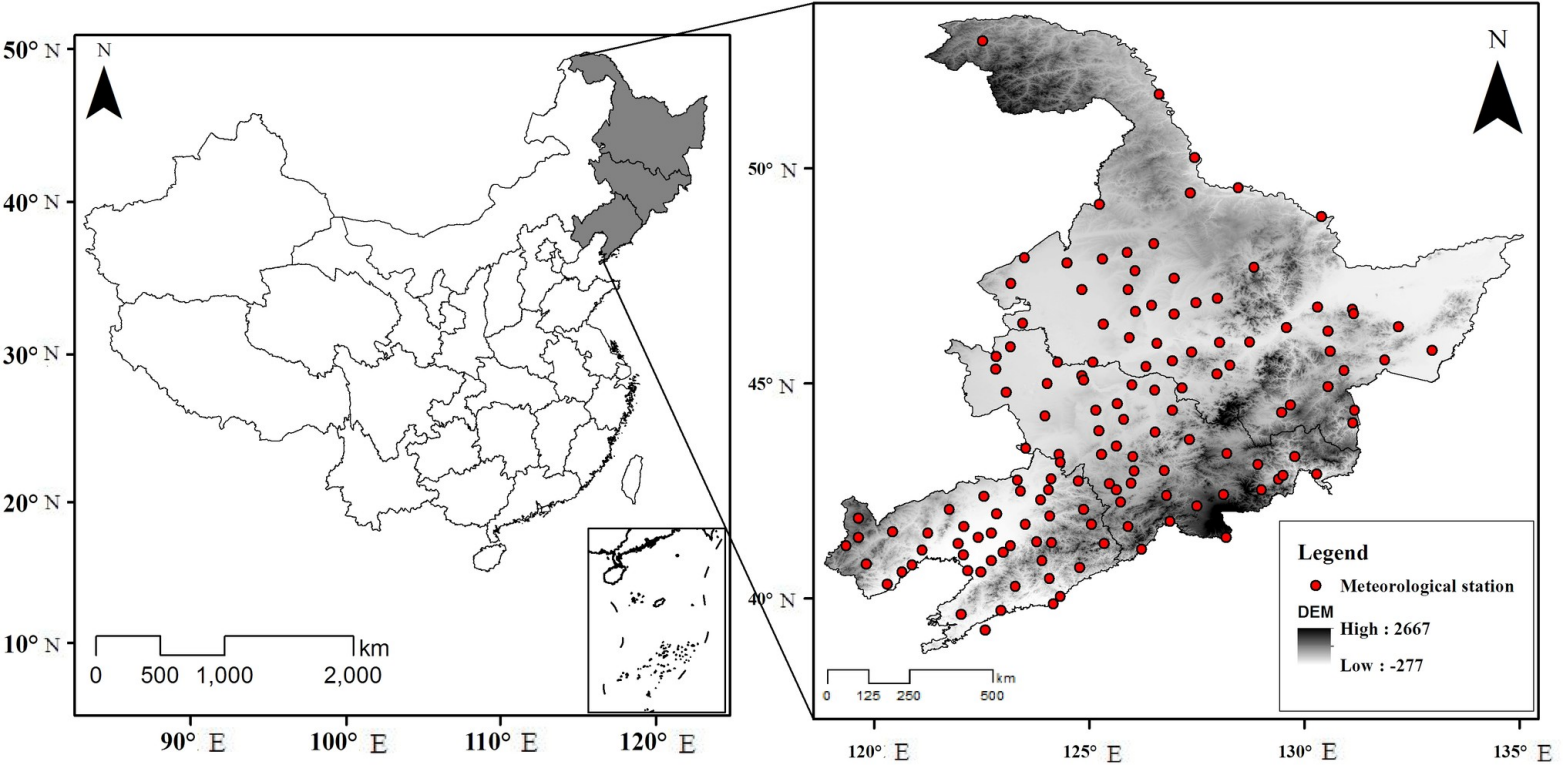
Map of study area and location of meteorological stations.

Data recorded at 140 meteorological stations during the 45-year period 1970–2014, including daily precipitation, average, maximum and minimum temperatures, wind speed, sunshine duration, and relative humidity, were provided by the China meteorological data service center (http://data.cma.cn/). The station locations are shown in Fig 1. The daily time series dataset is used to derive monthly values and to calculate potential evapotranspiration. The circulation indices, which were downloaded from the NOAA Climate Prediction Center (https://www.cpc.ncep.noaa.gov/), include the Southern Oscillation Index (SOI), POL, AMO, and the Arctic Oscillation (AO) Index.

### Potential evapotranspiration

The Penman–Monteith (PM) equation [42] is widely used to calculate potential evapotranspiration (PET) and FAO recommends its use. It reflects the influence of PET on drought severity, thereby making PM-based SPEI suitable for use in identifying drought-related effects. Zhao et al. [43] improved the computation of SPEI by using the PM method to calculate PET, and found SPEI thus calculated to be more dependable in Northwest China. Therefore, PM is used in this study to calculate PET. The calculation is:

$$PET=\frac{0.408\Delta (R_{N}-G)+\gamma [900/(T+273)]U_{2}(e_{s}-e_{a})}{\Delta +\gamma (1+0.34U_{2})}$$ (1)

where *PET* is the potential evapotranspiration (mm·day^−1^), Δis the slope of the curve representing saturation vapor pressure vs temperature (kPa/°C), *R*_*n*_ is the net radiation at the surface (MJ·m^−2^·day^−1^), *G* is the soil heat flux density (MJ·m^−2^·day^−1^), *γ* is the psychrometric constant (kPa/°C), *T* is the mean daily air temperature at a height of 2 m (°C), *U*_2_ is the wind speed at a height of 2 m (m/s), *e*_*s*_ is the saturated vapor pressure (kPa), and *e*_*a*_ is the observed vapor pressure (kPa).

### Calculation of SPEI

SPEI is based on the difference between precipitation and potential evapotranspiration. It can be used to distinguish dry or wet spells and to describe the degree of dryness or wetness by normalizing the difference between PET and precipitation. The following paragraphs briefly describe the calculation of SPEI.

First, calculate the monthly difference between precipitation and PET for month *i*:

$$D_{i}=P_{i}-PET_{i}$$ (2)

where *P*_*i*_ and *PET*_*i*_ are respectively precipitation and potential evapotranspiration for the *i*th month (mm).

Second, calculate the accumulated difference between precipitation and PET at different time scales (3-month, 6-month, 12-month, etc.). The accumulated difference (*X*^*t*^_*j*,*k*_) at the *t*-month time scale is calculated using the equation:

$$X_{j,k}^{t}=\sum_{j=i-t+1}^{j=i}D_{j,k}$$ (3)

where: *X*^*t*^_*j*,*k*_ is the accumulated difference between precipitation and PET at the *t*-month time scale in the *j*th month of the *k*th year; and *D*_*j*,*k*_ is the monthly difference between precipitation and PET in the *j*th month of the *k*th year.

Then use the three-parameter log-logistic probability distribution function to calculate the value of SPEI. The cumulative function of the log-logistic probability distribution F(*X*) is as follows:

$$F(X)={\left[1+{\left(\frac{\alpha }{X-\gamma }\right)}^{\beta }\right]}^{-1}$$ (4)

where *α*, *β*, and *γ* are scale, shape and position parameters, respectively.

The probability of a particular $X_{j,l}^{t}$ value, *p*, is given by:

p=1−F(X) (5)

If *p*≤0.5,

$$w=\sqrt{-2\ln p}$$ (6)

$$SPEI=w-\frac{C_{0}+C_{1}w+C_{2}w^{2}}{1+d_{1}w+d_{2}w^{2}+d_{3}w^{3}}$$ (7)

If p≥0.5,

$$w=\sqrt{-2\ln(1-p)}$$ (8)

$$SPEI=\frac{C_{0}+C_{1}w+C_{2}w^{2}}{1+d_{1}w+d_{2}w^{2}+d_{3}w^{3}}-w$$ (9)

where *C*_0_ = 2.515517, *C*_1_ = 0.802853, *C*_2_ = 0.010328, *d*_1_ = 1.432788, *d*_2_ = 0.189269, and *d*_3_ = 0.001308. Table 1 shows the threshold values of SPEI used to classify drought severity. In this study, the SPEI index at different time scales are used to analysis the seasonal and annual drought. For the Northeast China, spring is from March to May, summer is from June to August, autumn is from September to November, winter is from December to February.

**Table 1:** Drought severity classification according to SPEI values.

SPEI values	Drought classification
−0.5 < SPEI	No drought
−1.0 < SPEI ≤ −0.5	Light drought
−1.5 < SPEI ≤ −1.0	Moderate drought
−2.0 < SPEI ≤−1.5	Severe drought
SPEI ≤ −2.0	Extreme drought

### Determination of regions with homogeneous precipitation and PET patterns

Cluster analysis is widely used in climatology to divide a large area into homogeneous smaller regions based on climate variables. Because there are many climate types in the study area, we used the hierarchical clustering method to define different clusters of meteorological stations, based on annual precipitation and potential evapotranspiration. The combination of climate variables (precipitation and potential evapotranspiration) in each station is considered as a separate cluster and then clusters are compared. The clusters with the smallest between cluster dissimilarities merged until the desired numbers of clusters is reached. The Ward’s method with squared Euclidean distances measure is used to determine the clusters in this study.

### Drought trend analysis and correlation between drought and atmospheric circulation

The Mann–Kendall test (MK) and Sen’s slope estimator were used to estimate trends of drought events. The advantages of MK are that the data series are not required to fit any particular sample distribution and the sample data are assumed to be serially independent. Nonparametric MK has been widely used for trend analysis of time series [6, 44, 45]. The MK equation is:

$$S=\sum_{k=1}^{n-1}\sum_{j=k+1}^{n}sign(x_{j}-x_{k})$$ (10)

where *sign* is the sign function, and is:

$$sign(x_{j}-x_{k})=\{\begin{array}{ll} +1 & x_{j}-x_{k}>0\\ 0 & x_{j}-x_{k}=0\\ -1 & x_{j}-x_{k}<0 \end{array}$$ (11)

$$Var(S)=\frac{[n(n-1)(2n+5)]-\sum_{i=1}^{m}e_{i}(e_{i}-1)(2e_{i}+5)}{18}$$ (12)

where *n* represents the number of data points, *e*_*i*_ is the number of ties for the *i* tied value, and *m* is the total number of tied values. The *Z*-statistic can be calculated by:

$$Z=\{\begin{array}{ll} \frac{S-1}{\sqrt{Var(S)}} & S>0\\ 0 & S=0\\ \frac{S+1}{\sqrt{Var(S)}} & S<0 \end{array}$$ (13)

A positive value of *Z* represents an increasing trend and a negative value of *Z* represents a decreasing trend. If the *Z*-statistic reaches 1.65, 1.96, or 2.58, then the trend passes the significance test at the significance level of 90%, 95%, or 99%, respectively.

Sen’s slope method [46] is a simple nonparametric test to estimate the slope of the trend line. The method uses a linear model to determine the slope and Sen’s slope estimator (denoted by *Q*_*med*_) is calculated to show the trend of variation in the data. The details of the calculation can be found in Sen (1968) [46]. In this study, MK and Sen’s slope estimator were used to analyze the SPEI-12 time series and four seasonal (SPEI-3) time series for the 140 meteorological stations over the period 1970–2014 in order to detect trends in the variability of inter-annual drought in the study region. We looked for a significant upward or downward trend at the 90%, 95%, and 99% significance levels or a nonsignificant trend as shown by the *Z*-statistic values given by MK and the *Q*_med_ values of Sen’s slope estimator.

### Wavelet analysis

Wavelet analysis is used to analyze cycles in a time series. The cycles may have different period lengths which can vary with time. In general, the time series of climate variables is analyzed in terms of frequency. Wavelet analysis has the advantages of preserving local, non-periodic, and multi-scaled features when compared with conventional Fourier analysis. Wavelet analysis has become recognized as an efficient method to study intermittent localized oscillations.

The continuous wavelet transform (CWT) can be used to divide the time series into wavelets. It has been widely used to study the periodicity of droughts and has provided some important results [34, 47–49]. The continuous Morlet wavelet transform was chosen to study the periodicity of the drought series because it can well balance time and frequency localizations. The characteristic period of oscillation can be determined from the wavelet power spectrum. Because the time series are of finite length, the cone of influence (COI) was used to highlight the region of the wavelet spectrum where edge effects become important and results should be ignored [50]. Red noise was selected as the background power spectrum to examine the statistical significance at the 95% level since many geophysical time series have red noise characteristics.

The response of drought to different large-scale climate indices was examined using the cross wavelet transform (XWT) and wavelet coherence (WCO). The cross wavelet transform method is a multi-signal and multi-scale analysis technique based on traditional wavelet analysis [51]. It can be used to analyze correlation between two different time series and represents the phase structures and detailed features of wavelets in the time and frequency domains. WCO is used to estimate the intensity of the covariance and can be used to analyze the coherence and phase lag between two different time series as functions of both time and frequency. The statistical significance of the coherence was determined using Monte Carlo methods with red noise [52]. Both XWT and WCO have been widely used in climate research [6, 9, 49, 52].

## Results

### Trend detection

**Fig 2:**
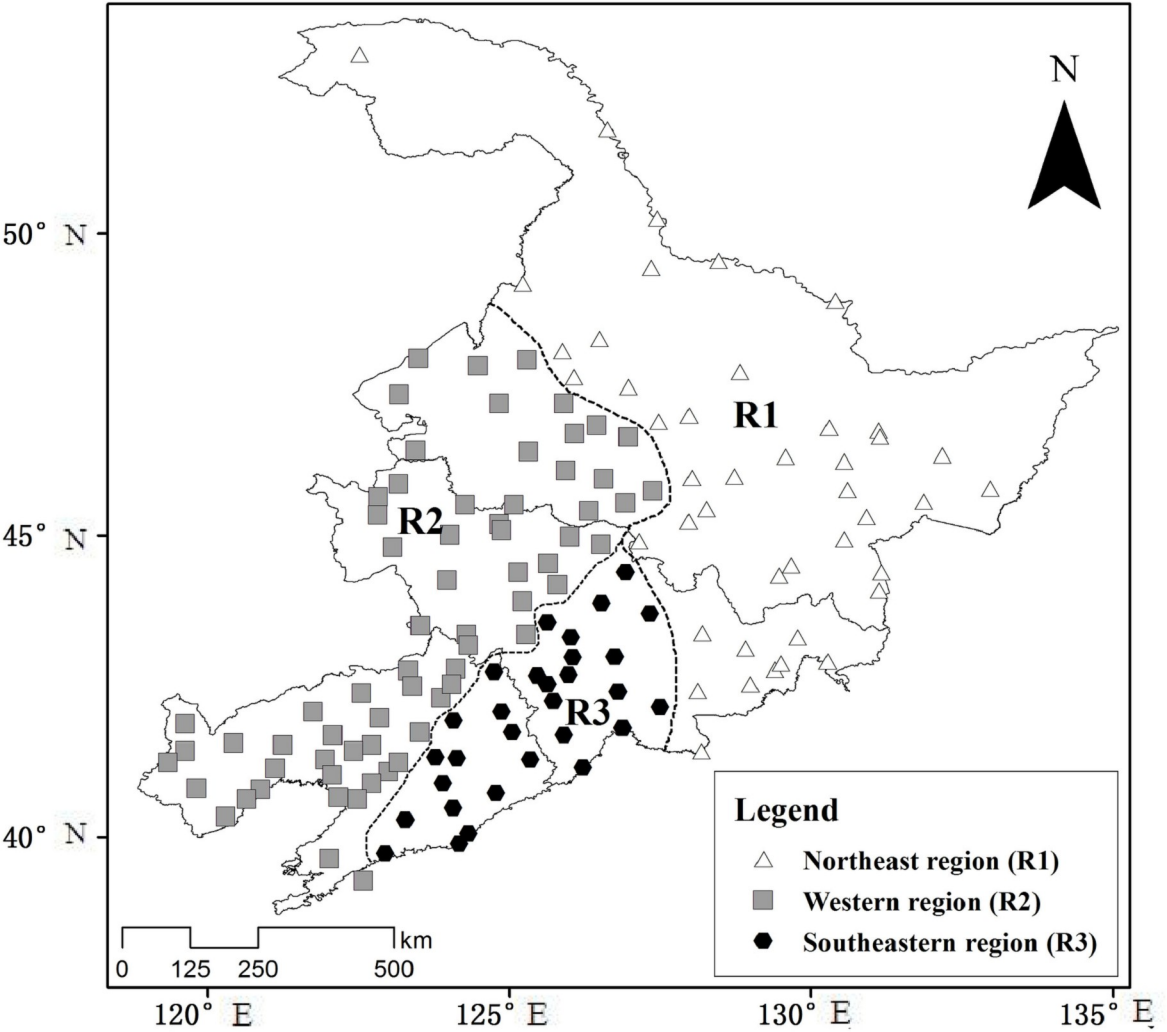
Partitioned results of cluster analysis.

Fig 2 shows the results of the cluster analysis of the study area. The three subdivisions are the northeastern region of Northeast China (R1), the western region (R2), and the southeastern region (R3). We analyzed the variation and distribution of drought in these three regions based on annual (SPEI-12) and seasonal (SPEI-3) SPEI values.

**Fig 3:**
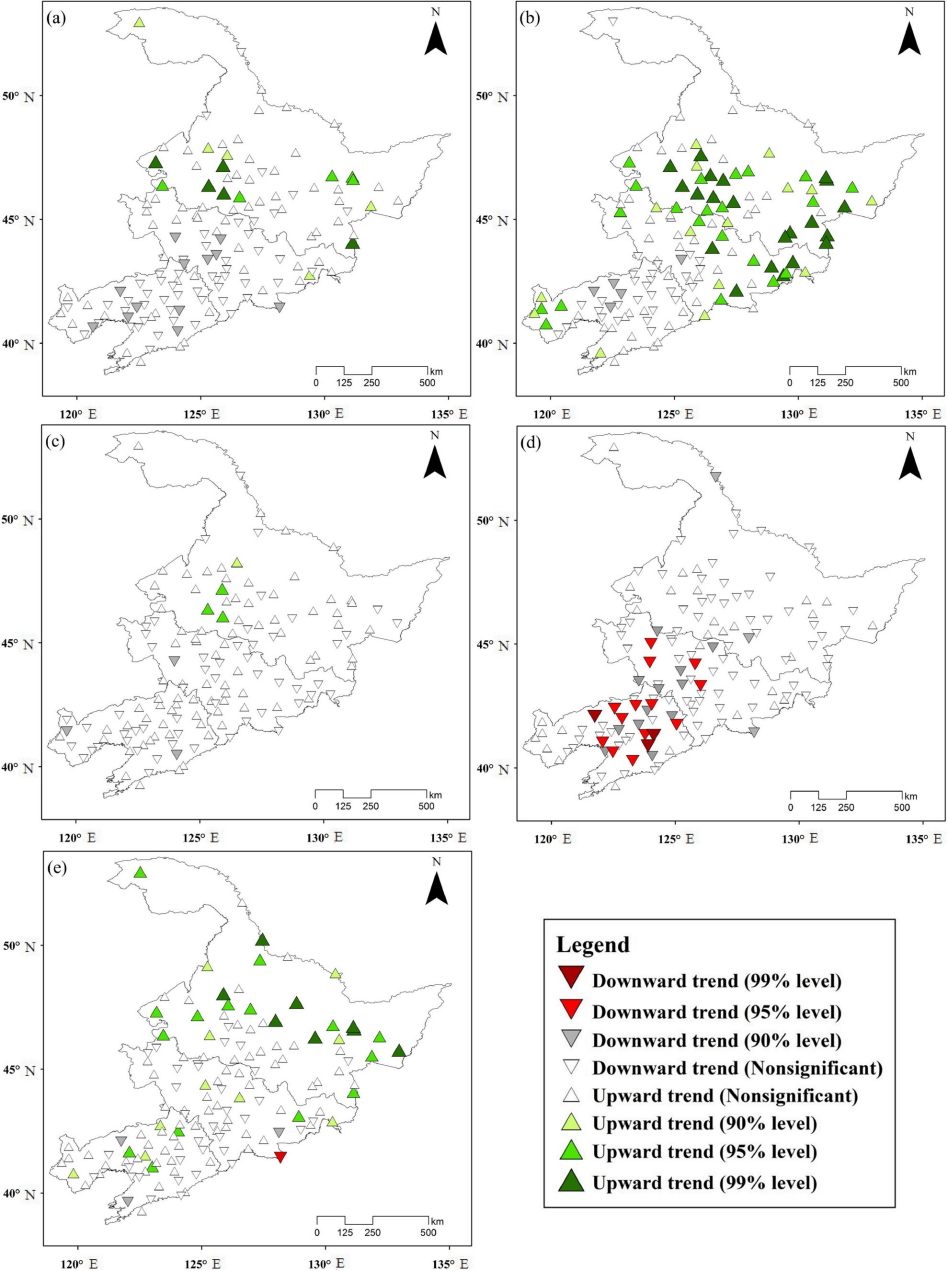
Spatial distributions for the trends of annual and seasonal SPEIs during 1970–2014: (a) annual; (b) spring; (c) summer; (d) autumn; (e) winter.

Fig 3 shows the spatial distributions of trends for the annual and seasonal SPEI time series of each station. Fig 3A shows that significant upward trends in the annual (SPEI-12) SPEI time series were detected at the stations in Heilongjiang province and the eastern part of Jilin province, which is mainly located in region R1. Significant downward trends were found in central Jilin province and Liaoning province, in regions R2 and R3. Nonsignificant upward trends were found mainly in region R1 and nonsignificant downward trends appear mostly in regions R2 and R3. These results indicate that inter-annual droughts have intensified in regions R2 and R3 but were mitigated in region R1. Region R1 includes Xiao Hinggan Mountains and Changbai Mountains, with more precipitation than other regions. Moreover, the latitude of R1 region is higher, the temperature is lower and the annual evapotranspiration is smaller. They are the reasons for reduced inter annual drought intensity in region R1.

Fig 3B shows that there was an increasing trend in the spring SPEI time series for more than 40% of all stations, most of which are located in region R1 (27 stations), region R2 (17 stations), and region R3 (6 stations). The increasing trend was significant in both western and eastern parts of Heilongjiang province and the eastern part of Jilin province, in regions R1 and R2. A significant decreasing trend appeared in three stations located in region R2. These results suggest an overall mitigation of drought in spring over Northeast China.

Fig 3C shows that there is no significant trend in the summer SPEI time series over most of the study area. It also suggests that there has been little change in summer drought during the 45-year study period.

Fig 3D shows a downward trend in the most parts of the study area. The significant downward trend in the autumn SPEI time series is seen in stations that are mainly located in the southwest of the study area, showing an increase in autumn drought in regions R2 and R3. This indicates an aggravated trend of autumn drought in study area, and a particularly significant aggravated trend in regions R2 and R3.

Fig 3E shows that the significant upward trends for winter SPEI time series are seen mainly in the northern part of the study area and at some individual stations in the southwest. The downward trends are mainly distributed in regions R2 and R3 but most are nonsignificant. These results show a mitigation of drought in winter in region R1.

### Periodic features of drought condition

**Fig 4:**
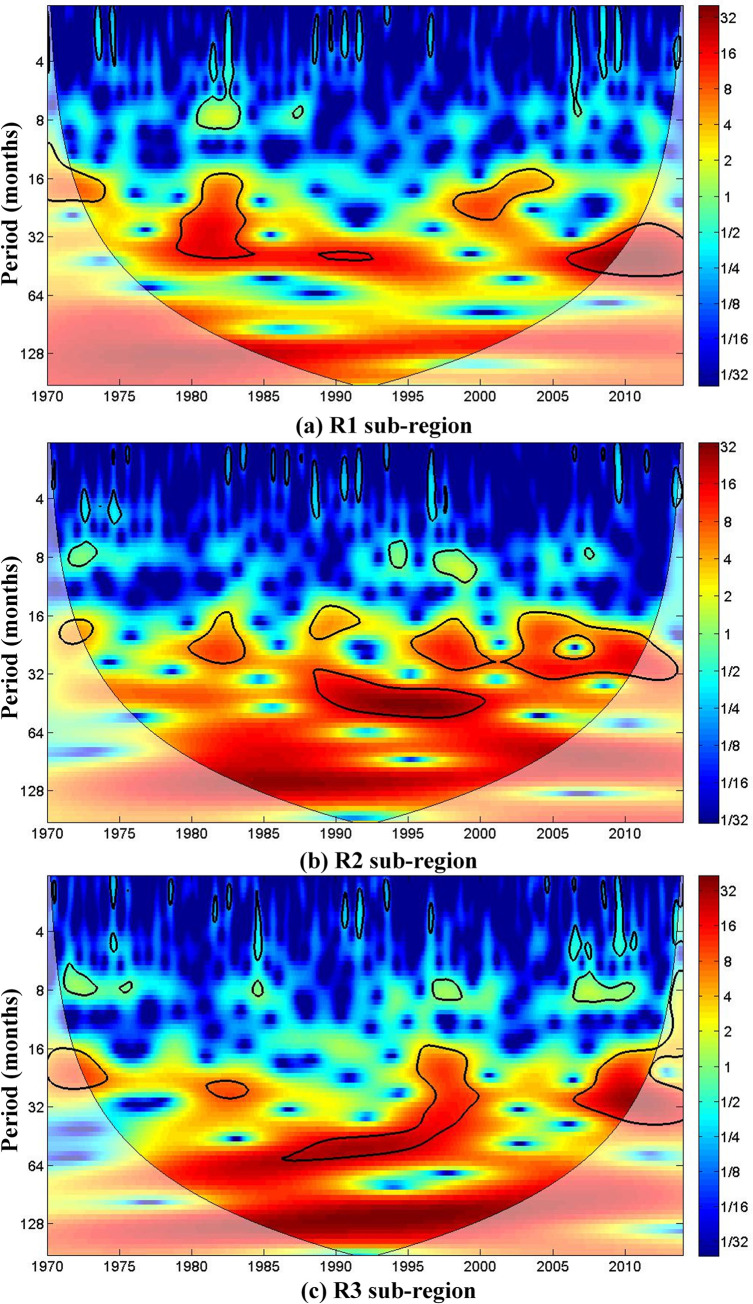
Continuous wavelet power spectrum of SPEI-12 series for the three regions. The thick black contours show the 5% significance level of local power relative to red noise. The cone of influence (COI), where edge effects are not negligible, is shown in a lighter shade.

CWT was used to analyze the periodic cycles at the 95% significance level in the three regional SPEI time series. The analysis of periodic characteristics based on CWT is conducted on the SPEI time series at 12-month scale (SPEI-12) rather than a 3-month scale so that the analysis is not affected by seasonal cycles. Fig 4 shows the wavelet power spectra (WPS) of SPEI-12 for the three regions in the study. As shown in Fig 4, there are several different cycles in the SPEI-12 series over the three regions which change in power over time.

The WPS of the SPEI-12 time series in region R1 shows continuous 15- to 42-month cycles of significant power in 1979–1984 and 1999–2005, and a 40- to 42-month cycle in 1987–1992 (Fig 4A). The long relatively high power 40- to 42-month cycles indicate that the drought effects in region R1 were not significant; they occurred mainly in the 1970s, 1980s and the most recent ten years.

The WPS of the SPEI-12 time series for region R2 shows 5 significant cycles of relatively high power during the study period (Fig 4B). The 14- to 30-month cycles show significant power in the periods 1980–1984, 1987–1991, 1996–2002 and 2004–2014. The 30- to 55-month cycle shows significant high power in 1988–2000. These cycles may strongly influence the development of drought events in region R2.

The WPS of the SPEI-12 time series for region R3 shows a significant 23- to 30-month cycle in 1981–1984, as well as the significant15- to 60-month cycle in 1986–1999 (Fig 4C). The 16- to 40-month cycle of significant power in 2006–2014 can account for the intensification of droughts over region R3 over the last ten years. The climate oscillation with high power around the 128-month can be observed over each of the three regions, but there are differences in energy between the three regions. The power of oscillation in region R3 is higher than in the other two regions.

The variability of drought in Northeast China is shown in the differences in the cycles related to the SPEI-12 time series between the three regions. The similar time scales of the cycles show the coordinated development of drought in the three regions, but cycles in regions R2 and R3 have greater power than those in region R1. These differences confirm that the temporal characteristics of droughts differ between the regions. Conclusions about periodic features in this study are restricted to an inter-annual scale because of the temporal shortness of the data series. However, the different cycles shown by CWT can provide useful information for drought risk analysis in the different regions and will lead to a better understanding of the atmospheric dynamics that modulate drought occurrence and severity on this regional scale.

**Fig 5:**
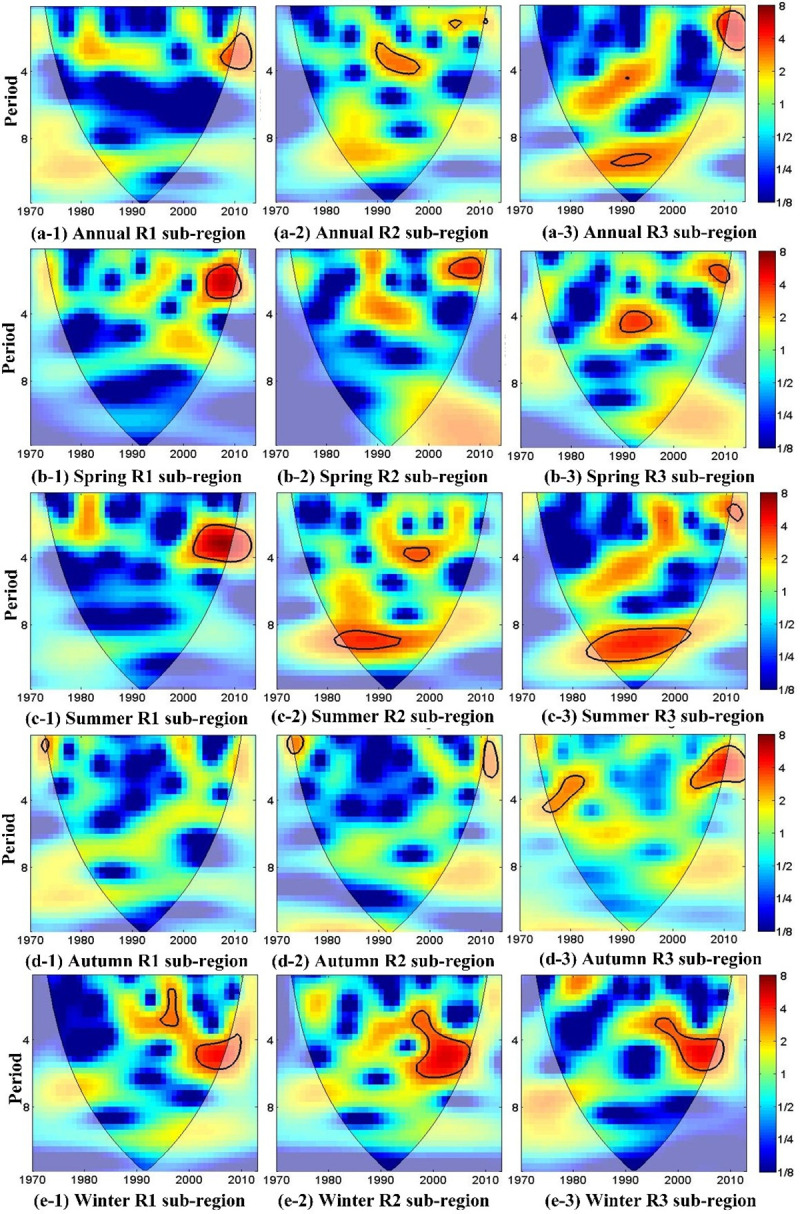
Continuous wavelet power spectrum of annual and seasonal SPEI series in the three regions. The thick black contours depict the 5% significance level of local power relative to red noise; the cone of influence (COI), where edge effects are not negligible, is shown as a lighter shade.

CWT was also used to analyze the periodic characteristics of annual and seasonal SPEI time series in an attempt to identify the periodic features of annual and seasonal droughts in each of the three regions, as shown in Fig 5.

The WPS for the annual SPEI time series for region R1 (Fig 5A-1), the 2- to 4-year cycles show significant power after 2005.The WPS for the annual SPEI time series in region R2 (Fig 5A-2) shows that 2.5- to 3.5-year cycles have significant power in 1989–1999, and a quasi-biennial periodic structure can be detected in 2003–2006. The WPS for the annual SPEI time series in region R3 (Fig 5A-3) shows two significant cycles with different time scales. The 9- to 10-year cycle shows significant power in 1987–1996 and the 2- to 4-year cycle does so in 2003–2011. This suggests that the annual drought in Northeast China is mainly characterized by 2- to 4-year cycles. The periodic analysis results of annual SPEI are basically consistent with the above results for the SPEI-12 series.

Fig 5B–5E show WPS for the seasonal SPEIs (SPEI-3). Significant wavelet power at the 2- to 6-year scale can be seen for all three regions, although the specific periodic patterns vary from season to season, depending on the region. The WPS for autumn SPEI series is minimum. The power intensities of cycles in regions R2 and R3 are higher than those in region R1. These periodic analysis results imply that droughts in the study area seem to be related to different oscillations in the global climate system. The dominant climate factors also change spatially over the study area, corresponding with the three regions that we identified, which have distinct temporal drought development conditions.

### Correlations between atmospheric circulation and drought condition

XWT and WCO were used to identify common power intensities and correlations between the climate indices for the teleconnections ENSO, AMO, AO and POL and regional drought severity, which is represented by the average of SPEI-12 for each of the three regions. The identifications are made on inter-annual and decadal scales at the ≥95% significance level.

**Fig 6:**
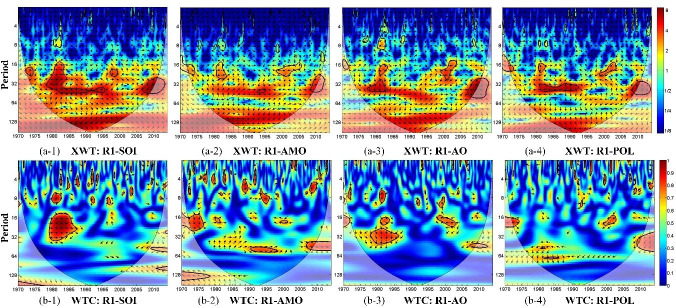
(a) Cross wavelet power spectrum (XWT) and (b) wavelet coherence (WTC) between SPEI-12 over region R1 and climate indices. The thick black contours depict the 5% significance level of local power relative to red noise; the cone of influence (COI) is presented as a lighter shade. Right-pointing arrows indicate that the two signals are in phase while left-pointing arrows show anti-phase signals.

Fig 6 shows the cross-wavelet power spectrum and wavelet coherence for SPEI-12 and each climate index in region R1. The figure shows that drought in region R1 is significantly correlated with SOI in the 14- to 40-month period in 1979–1987; with the AMO index in the 14- to 27-month period in 1972–1977 as well as in the 54- to 60-month period in 1991–1998; with the AO index in the 24- to 43-month period in 1977–1986 and in the 15- to 23-month period in 1998–2004; and with the POL index in the 15- to 22-month period in 1997–2003. These correlations show the significant influence of the climate factors on drought conditions in region R1 on an inter-annual scale.

**Fig 7:**
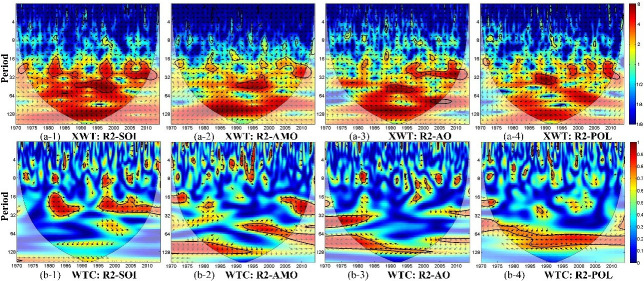
(a) Cross wavelet power spectrum (XWT) and (b) wavelet coherence (WTC) between SPEI-12 time series for region R2 and climate indices. The thick black contours depict the 5% significance level of local power relative to red noise; the cone of influence (COI) is shown as a lighter shade. Right-pointing arrows indicate that the two signals are in phase while left-pointing arrows show anti-phase signals.

Fig 7 shows the cross-wavelet power spectrum and wavelet coherence for SPEI-12 and each climate index in region R2. The figure shows that drought in region R2 is significantly correlated with SOI in the 14- to 35-month period in 1979–1989 and in the 16- to 30-month period in 1996–2003; with the AMO index in the 43- to 76-month period in 1992–2007 and in the 16- to 32-month period in 2004–2013; with the AO index in the 24- to 43-month period in 1973–1983 and in the 65- to 128-month period in 1977–2001; and with the POL index in the 42- to 107-month period in 1975–2007. These correlations show the significant influence of climate factors on drought conditions in region R2 on an inter-annual scale.

**Fig 8:**
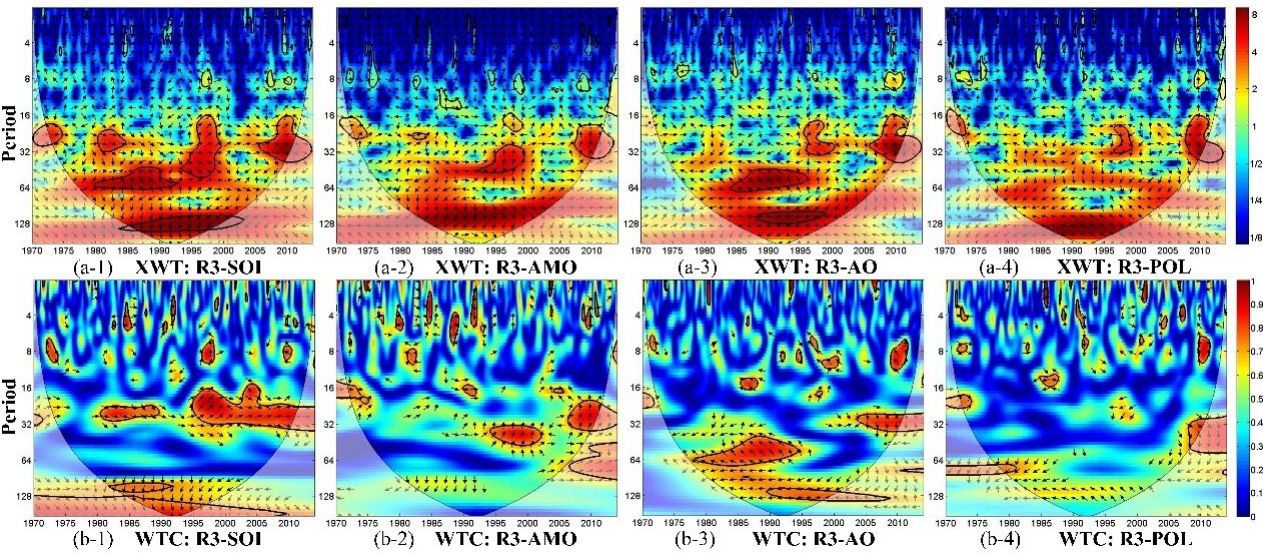
(a) Cross wavelet power spectrum (XWT) and (b) wavelet coherence (WTC) between SPEI-12 over region R3 and climate indices. The thick black contours depict the 5% significance level of local power relative to red noise; the cone of influence (COI) is shown as a lighter shade. Right-pointing arrows indicate that the two signals are in phase while left-pointing arrows show anti-phase signals.

Fig 8 shows the cross-wavelet power spectrum and wavelet coherence for SPEI-12 and each climate index in region R3. The figure shows that drought in region R3 is significantly correlated with SOI in the 22- to 31-month period and in the 98- to 108-month period in 1981–1991 and in the 14- to 35-month period in 1994–2011; and with the AO index in the 31- to 73-month period in 1977–1994 and in the quasi-128 month cycle in 1989–2001. The AMO index is strongly correlated with drought conditions in region R3 in the 32- to 50-month period in 1993–2002. Drought variability in region R3 is weakly correlated with the Polar–Eurasia pattern (represented by the POL index) at a scale >1 year.

We conclude that the global teleconnections (including ENSO, AMO, AO, and POL) generally all strongly influence drought in Northeast China, concentrated in the periods that were identified in our analysis, but at different time scales. However, these influences vary in the three distinct regions of the study area. The influence of the connections between large scale climate patterns and drought conditions in regions R2 and R3 is greater than in region R1. It is basically consistent with the potential long-term periodicity observed for regional drought suggested by continuous-wavelet transform analysis that large-scale climate patterns may play a crucial role in the periodical nature of drought occurrences. It is also notable that SOI and AO show possible connections to drought variability on inter-annual scales.

Cross-wavelet analysis facilitated the investigation of connections between the climatic teleconnections and annual and seasonal droughts. To analysis seasonal drought (represented by seasonal SPEI values), the index value for each climate pattern considered in the analysis was defined as the average value of its index for the months in that season. Table 2 summarizes the significant correlations between each climate index and the annual or seasonal SPEI values for each of the three different regions, according to the common power and wavelet coherence calculated by the XWT and WTC methods.

**Table 2:** Summary of significant correlations between each climate index and annual and seasonal SPEIs over different regions based on the results of XWT and WCO.

SPEI	Climate Index	Region R1		Region R2		Region R3	
		Time	Period (years)	Time	Period (years)	Time	Period (years)
Annual	SOI	—	—	1996–2002	2–4	1995–2000	2–4
	AMO	1981–1988	2–4	1978–1998	7–10	1992–2004	2–4
		1992–1999	3.5–5	1993–2006	3–6	2006–2011	2–4
	AO	1976–1984	2–4	1973–1983	2–4	1976–1996	2–6
				1978–1989	5–8	2005–2011	~2
	POL	1979–1986	2–4	1975–1984	2–4	1979–1986	5–7
				1990–2005	6–8		
Spring	SOI	1976–1988	3–5	1980–1985	3–4	—	—
		1988–2007	6–8	2002–2006	5–6		
	AMO	1982–1987	2–5	1977–1994	2–4	1989–1997	2–4
				1984–2005	7–8		
	AO	1980–2000	7.5–10	1980–2000	7.5–9	1980–2003	7.5–9
				2005–2010	2–4	2002–2008	2–4
	POL	1983–2007	5–7	1982–1997	2–4.5	1988–1993	2–4
Summer	SOI	—	—	—	—	1982–1995	8–10
						1993–2001	~2
	AMO	—	—	1975–1987	2–4	1980–2001	7–10
				1981–2000	7.5–10		
				1994–2000	3.5–5		
	AO	1983–1990	8–10	1978–1985	4.5–6	1976–1996	4–6
				1993–1997	~2		
	POL	1973–1985	2–4	1983–1995	5–7	—	—
				1991–1997	2–5		
Autumn	SOI	1985–1989	~2	1991–2001	3–7	1991–1999	4.5–6
	AMO	—	—	—	—	—	—
	AO	1977–1987	2–5	1997–2003	4–6	1999–2005	4.5–6
	POL	2005–2009	2–4	1981–1985	2–4	1981–1989	2–4
				1988–2008	2–6	1997–2008	2–6
Winter	SOI	1998–2001	2–4	1991–2000	9–12	1996–2001	3–4
		1987–2001	12–15				
	AMO	1990–2001	2–5	1989–2001	2–5	1985–2002	2–5
	AO	—	—	1985–1991	3.5–5	—	—
	POL	1985–1995	2–5	1980–2010	2–7	1986–1991	2–4
		1978–2009	3–7			2002–2009	2–7

Table 2 shows that the annual drought variability in region R1 is significantly correlated with: the AMO index in a 2- to 4-year period in 1981–1988 and a 3.5- to 5-year period in 1992–1999; the AO index in the 2- to 4-year period in 1976–1984; and POL in the 2- to 4-year period in 1979–1986.

The annual drought variability in region R2 is significantly correlated with (p<0.05): SOI in a 2- to 4-year period in 1996–2002; the AMO index in a 7- to 10-year period in 1978–1998, and in a 3- to 6-year period in 1993–2006; the AO index in a 2- to 4-year period in 1973–1983, and in a 5- to 8-year period in 1978–1989; and the POL index in a 2- to 4-year period in 1979–1986.

Drought in region R3 oscillates and is significantly correlated with: both SOI and the AMO index with a 2- to 4-year period in 1992–2004 and 2006–2001; the AO index with a 2- to 6-year period in 1976–1996 and ~2-year period in 2005–2011; and the POL index with 5–7 year period in 1979–1986.

Overall, these climate patterns all have significant influence on spring drought in Northeast China, mainly in the 2- to 5-year period and in the 7- to 10-year period. AMO has no significant influence over region R3. There is a significant association with AO in the 4- to 6-year period for all three regions for summer drought. There is very strong association with AMO and POL in region R2, and with AO in region R1. There is clear association with SOI and AMO mainly in the 7- to 10-year period in region R3 for summer drought. Variability in the autumn drought is significantly correlated with SOI, the AO index, and the POL index in the 2- to 6-year period across the whole area (p<0.05). There is no statistically significant influence of AMO on autumn drought. Winter drought conditions over the region are influenced by SOI, AMO, and POL in 2- to 7-year periods. The associations between AMO and drought conditions are anti-phase. SOI is significantly correlated in region R1 in a 12- to 15-year period and in region R2 in a 9- to 12-year period.

## Discussion and conclusions

According to previous analysis, we found that the spring and winter droughts are alleviated, especially in region R1, and the autumn drought in region R2 and R3 showed a significant increasing trend. The variation in summer drought is alleviated but the trend is not significant. The different trends of precipitation directly affect the variation of drought in different sub regions of Northeast China. The precipitation variability of northeast China is large, and the monthly precipitation of northeast China varied considerably. Precipitation is concentrated in summer, the summer precipitation accounted for 65.7% of the annual total value [53]. The seasonal precipitation showed various trends, the spring precipitation and winter precipitation showed increasing trend, the summer precipitation and autumn precipitation showed decreasing trend. The trend of summer precipitation is not significant. The precipitation of Liaodong Peninsula (locate in the region R2) and southern Changbai Mountain (locate in the region R3) showed significantly decreasing trend. The precipitation variation of the northern Da Hinggan Mountains (locate in the region R1) showed obviously increasing trend [54]. The increasing trend of precipitation in spring and winter leads to the relief of drought in Northeast China, while the decrease of precipitation in autumn is obvious, especially in R2 and R3 sub regions, which also aggravates the threat of autumn drought in the above regions. The trend of precipitation in summer is not obvious. With the northward movement of subtropical high, the precipitation in Northeast China is concentrated in summer, so the trend of drought in summer is not obvious. Their studies further support my analysis. The similar results were found in Nigeria, the results showed that drought severity has increased during the cropping seasons [55].

Global warming has increased the complexity of identifying the causes of drought variability in Northeast China. Previous studies have shown the significant effects of large-scale climate patterns on drought. In this study, we used cross-wavelet analysis to determine the correlation between large-scale climate patterns and drought conditions in the time–frequency domain. The results explain the differences in drought events between the different regions and they suggest that different climate patterns affect drought conditions at various time scales. And the relationships between large-scale climate patterns and drought can also be used as the basis for constructing drought prediction model based on machine learning method in the future [56].

ENSO is an irregularly periodic variation in winds and sea surface temperatures over the tropical eastern Pacific Ocean. It has a great impact on global climate change. There is a significant influence of ENSO on the variation of SPEI in Chile [57].The variation of ENSO can affect rainfall in Northern China and the development of drought. The Southern Oscillation is the atmospheric component of El Niño and SOI can be used to describe ENSO. Negative SOI indicates El Niño episodes and positive SOI indicates La Niña. During the typical warm phase of ENSO (with negative SOI), there are higher temperatures and less precipitation in Northern China and surface conditions become drier, leading to the occurrence of drought events [58, 59]. Luo [60] found that in the developing stage of ENSO, summer precipitation in Northern China is generally lower, possibly causing drought. Deng et al. [34] noted that ENSO events (which are represented by SOI) influenced summer drought in the middle–upper reaches of the Pearl River basin and strongly influenced the occurrence of autumn drought in the Pearl River Delta. Our findings that there are strong in-phase associations between ENSO and drought conditions in northeast China. There is a positive correlation between SPEI and SOI. This relationship indicates that during the El Niño event, the threat of drought will grow. Our analysis results are agree with the above research. The main reason for this is that the El Niño event will increase the abnormal change of atmosphere. Relevant studies suggest that northeast China is controlled by an extremely strong anticyclone in the developing stage of El Nino development period [61].

AMO is a climatic cycle that affects the sea surface temperature (SST) of the North Atlantic Ocean [62], but it also affects eastern Asia. AMO is conducive to East Asian climate warming, and the warm phase of AMO intensifies the East Asia summer monsoon and weakens the winter monsoon [63, 64]. We found that the influence of AMO on drought conditions in Northeast China varies seasonally. AMO has a stronger influence on drought conditions during winter and spring than in summer and autumn, especially on the inter-annual scale. Some studies indicate that AMO plays an important part in winter warming and precipitation in Northern China. Wang et al. [48, 63] found a significant correlation between AMO behavior and drought variability in the Luanhe River basin on different time scales, which is similar to the results of this study. Our results showed that the influence of the Atlantic Multidecadal Oscillation (AMO) on drought in Northeast China is concentrated in the inter-annual scales. The significant cycles mainly occurred in spring, summer and winter, and the associations between AMO and winter drought are anti-phase. This may be related to the effect of AMO change on precipitation. During the warm phase of AMO, there are more precipitation in spring, summer and autumn, winter precipitation in Northern China is generally lower. But the relationship between them is not significant in autumn.

AO dominates tropospheric circulation in the middle–high latitudes of the Northern Hemisphere [65]. The AO pattern produces negative correlations between the AO and SPEI in almost entire Iberian Peninsula [66].When AO is in a positive phase, pressure is low in the Arctic and high in the middle latitudes, so cold air is contained in the polar region. In the negative phase, the pattern is reversed, which allows cold polar air to move southward. The polar region is the main source of the cold air and the cold air activity is easily affected by AO. Thus AO has a significant influence on the East Asian climate. Wang et al. [67] found that there is a close positive correlation between the temperature in Northeast China and the AO index. Studies suggest that there is a significant relationship between AO and precipitation [68–71]. Qu et al. [72] noted that during the positive (negative) phase of AO, precipitation around East Asia tends to be less-than-normal (more-than-normal). Li et al. [73] found a significant correlation between AO behavior and drought in the Luanhe River basin. In this study, we found that AO strongly influenced drought conditions in Northeast China. There is a negative correlation between AO and drought evolution at different time scales. The correlation between AO and the East Asian trough is high. Associated with the positive AO, the East Asia trough, Siberian high are all weaker. In lower level, there is an anticyclonic circulation anomaly in the Northeast China. The anticyclonic circulation will lead to less precipitation, possibly causing drought [68]. The consistency between this study and the results of previous studies strengthens the credibility of our findings on the effect of AO.

The Polar–Eurasia pattern (POL) appears in all seasons. When POL is in a positive phase, pressure is low in the polar region and high in the north part of China, so cold air is contained in the polar region. In the negative phase, the pattern is reversed, which is not favorable for the southward motion of the cold air. POL represents the strength of circumpolar circulation. The positive phase of POL indicates the enhanced circumpolar vortex and the negative phase reflecting polar vortex weaken. During the positive phase of POL, the cold air will hard to spill southward, giving a positive height anomaly over Northern China. This atmospheric pressure anomaly and circulation are not conducive to precipitation and they increase the risk of drought in Northeast China. The other studies point out that there is a relationship between POL and atmospheric circulation over Asia [74]. The variation of POL will influence the westerly wind and the persistent vortex over Asia in summer, which leads to precipitation anomalies over this area. Ying et al. [75] also found that the POL pattern is strongly related to precipitation in Northeast China. Yue et al. [76] also found the relationship between POL and drought in Northeast China (represented by RDI) is stronger than the other climate patterns. Moreover, POL has obvious lagged effect on the variation of drought. Our findings that there are strong relationships between POL patterns and the climate of Northeast China agree with these conclusions. Our results show a significant relationship between POL and drought conditions at different time scales over Northeast China.

Monthly meteorological data from 140 stations in Northeast China observed during the period 1970–2014 were used to calculate the Standardized Precipitation Evapotranspiration Index (SPEI). Our estimate better models the effect of PET on drought severity and so makes SPEI even more suitable for identifying and analyzing the effects of drought in our research area. Hierarchical cluster analysis was used to define the spatial structure of regional climate based on the precipitation and potential evapotranspiration data. A well-defined spatial structure with three distinct regions was determined for Northeast China, namely the northeastern region (R1), the western region (R2), and the southeastern region (R3). The Mann-Kendall trend test and Sen’s slope estimator were used to identify the temporal trends in drought, and the cross wavelet transform was used to identify periodic features. Cross wavelet analysis enabled the identification of relationships between large climate patterns and drought variability in the three different regions. The main conclusions are summarized as follows:

1. Over Northeast China, the results of MK and Sen’s slope estimator generally show evidence of mitigation of spring drought and winter drought, mainly in region R1, together with a significant increasing trend in autumn drought, mainly in regions R2 and R3. The variation in summer drought is not significant. At an annual scale, drought severity intensified in regions R2 and R3 but was attenuated in R1.
2. Drought in Northeast China has definite periodicity. In terms of SPEI-12, drought conditions in the three regions are similar, having 14- to 42-month or 15- to 60-month cycles. Cycles in regions R2 and R3 are more powerful and more intense than in R1. There is clear evidence that annual and seasonal droughts have a 2- to 6-year cycle.
3. Large-scale climate patterns (ENSO, AMO, AO, and POL) have a significant influence on drought in Northeast China with a 14- to 30-month period. With regard to annual and seasonal scales the influence of large scale climate patterns on drought condition are mainly inter-annual and decadal. These patterns may be responsible for the periodic behavior of regional drought conditions. SOI, AO, and POL indexes are significantly correlated with drought variability on decadal scales (~120-month period).

Our results provide valuable information for understanding how drought characteristics vary and can be used to improve the planning and management of agricultural production in Northeast China. In addition, the likely correlation between the climate indices and the drought index can be used to improve the accuracy of drought forecasting. However, drought characteristics have mainly been derived from historical data. Since climate change is dynamic and a persistent phenomenon, there is a continuing need to further examine the effects of climate change on drought in Northeast China.

## Supporting information

S1 File (ZIP)
